# Lessons from temporal and spatial patterns in global use of N and P fertilizer on cropland

**DOI:** 10.1038/srep40366

**Published:** 2017-01-13

**Authors:** A. F. Bouwman, A. H. W. Beusen, L. Lassaletta, D. F. van Apeldoorn, H. J. M. van Grinsven, J. Zhang, M. K. Ittersum van

**Affiliations:** 1 Department of Earth Sciences - Geochemistry, Faculty of Geosciences, Utrecht University, P.O. Box 80021, 3508 TA Utrecht, The Netherlands; 2 PBL Netherlands Environmental Assessment Agency, PO Box 30314, 2500 GH The Hague, The Netherlands; 3 Farming Systems Ecology group, Wageningen University, PO Box 430, 6700AK Wageningen, The Netherlands; 4 Center for Earth System Science, Tsinghua University, 100084 Beijing, China; 5 Plant Production Systems Group, Wageningen University, P.O. Box 430, 6700 AK Wageningen, The Netherlands

## Abstract

In recent decades farmers in high-income countries and China and India have built up a large reserve of residual soil P in cropland. This reserve can now be used by crops, and in high-income countries the use of mineral P fertilizer has recently been decreasing with even negative soil P budgets in Europe. In contrast to P, much of N surpluses are emitted to the environment via air and water and large quantities of N are transported in aquifers with long travel times (decades and longer). N fertilizer use in high-income countries has not been decreasing in recent years; increasing N use efficiency and utilization of accumulated residual soil P allowed continued increases in crop yields. However, there are ecological risks associated with the legacy of excessive nutrient mobilization in the 1970s and 1980s. Landscapes have a memory for N and P; N concentrations in many rivers do not respond to increased agricultural N use efficiency, and European water quality is threatened by rapidly increasing N:P ratios. Developing countries can avoid such problems by integrated management of N, P and other nutrients accounting for residual soil P, while avoiding legacies associated with the type of past or continuing mismanagement of high-income countries, China and India.

Depletion of phosphate rock reserves and increasing environmental problems associated with inefficient use of phosphorus (P) and nitrogen (N) in agriculture have induced many new research efforts which mostly focused on either N or P^1^,^2^,^3^,^4^,^5^,^6^. Since in the absence of fertilization both nutrients are normally limiting agricultural crop production, and because of the large differences between the biogeochemical cycles of N and P, their joint study is essential to understand their limitation, surplus and balance^7^,^8^. During the time period 1970–2010, world consumption of mineral N fertilizers grew much more than that of mineral P fertilizers, leading to an increase of the molar N:P ratio in global fertilizer use from 8 to 13^9^,^10^. Four questions arise from this: (i) does the situation vary between countries that differ in their agricultural development stage; (ii) are these changes pointing to true nutrient imbalances; (iii) are there environmental impacts (river water quality) due to differential changes in N and P fertilizer use, and (iv) which lessons can be learnt from this for farm practice and policy? To answer these questions, we analyze the evolution of soil N and P cycles in crop production during the past decades (1970–2010) in different world regions, and discuss the differences in the light of the biogeochemical behavior of N and P.

In agronomy, nutrient cycles are often studied by comparing soil N and P budgets. Agronomic soil nutrient budgets are the difference between the sum of all inputs and crop withdrawal^3^,^6^. The N and P use efficiency (NUE, PUE) we use is defined as the ratio of output of N or P in harvested crop parts: input (including N inputs from fertilizer, manure, atmospheric deposition and biological N fixation, and P inputs from fertilizer and manure). Residual soil budgets are the accumulation of nutrients in soils over multiple seasons, which also account for other nutrient loss pathways, such as erosion. This is most applicable to P, as much of the residual N after one season will be leached, denitrified or taken up by the next crop. Here, we use spatially explicit data from the database used in the IMAGE-Global Nutrient Model^11^,^12^ to analyze the simultaneous changes in soil nutrient budgets, nutrient use efficiencies and residual soil P for all annual and perennial crops and fruits. Agronomic budgets of N were calculated for each 0.5 by 0.5 degree grid cell as the sum of biological fixation (legume crops), deposition, mineral fertilizer and manure, minus the N in the crop yields. For P the same approach was used, without the fixation and deposition components and including runoff losses to compute residual soil P changes (see methods). The consequences of changing nutrient budgets in agricultural systems are discussed to draw lessons for developing countries that are still in an early phase of agricultural development. We do not present the loss pathways for N and P and the fate of these nutrients as these have been presented in a series of papers on gaseous N losses^13^,^14^ and the fate of nutrients in inland waters^11^,^12^.

## Results and Discussion

### Spatial and temporal patterns in N and P use

**Figure 1:**
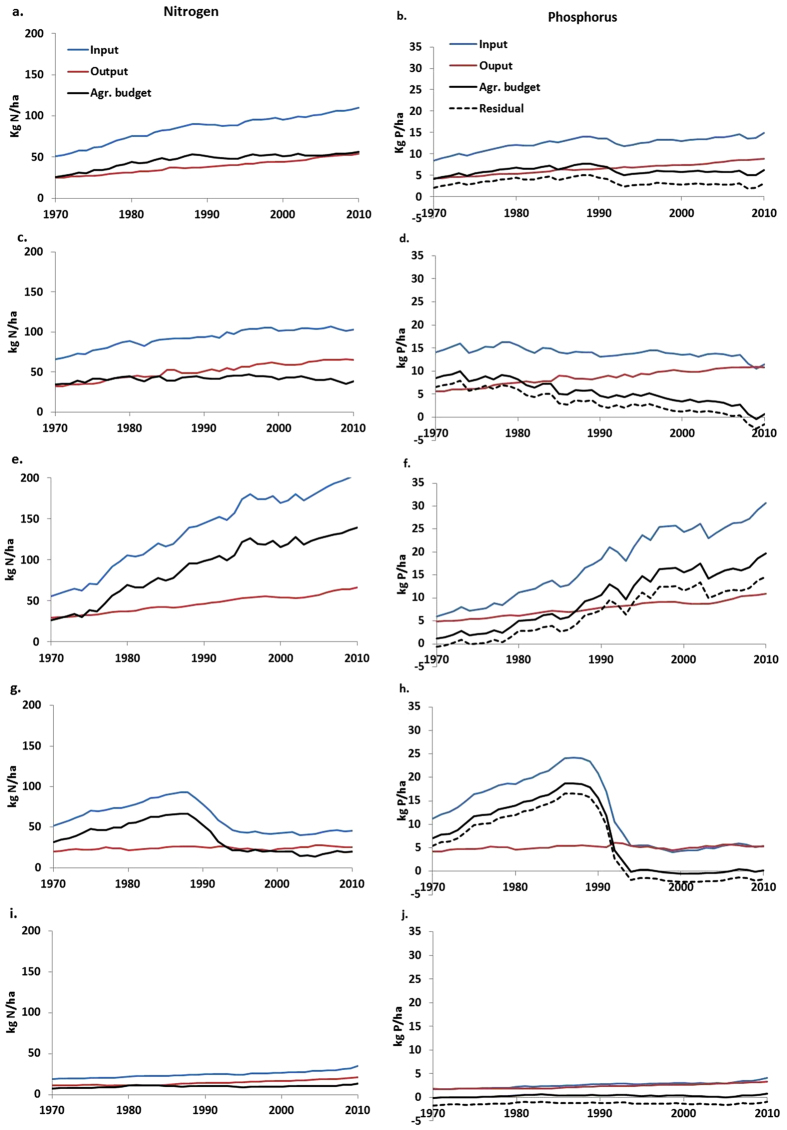
Inputs, outputs and agronomic soil budgets of N (left) and P (right) for cropland. Inputs (including fertilizer, manure, biological N fixation, N deposition), outputs (including N and P withdrawal in harvested parts of crops), and agronomic soil budget (difference between inputs and outputs) per hectare of cropland for N (left) and P (right) from 1970 to 2010 for the world (**a**,**b**), high-income countries (USA, Canada, Western Europe, Japan, Australia, New Zealand) (**c**,**d**), China and India (**e**,**f**), transition countries (Eastern Europe and former Soviet Union) and sub-Saharan Africa (Africa excluding Algeria, Egypt, Libya, Morocco, Tunisia, Western Sahara and South Africa) (**g**,**h**) (**i**,**j**). The graphs for P also show the residual P, which is the agronomic soil budget minus runoff losses. Data on inputs and outputs for 26 world regions (and SI Datasets; region definition, see Table SI1) as well as grid maps of P inputs and outputs needed to compute the residual P budget for 2010 (Figure SI5) are available in the Supplementary Information.

**Figure 2:**
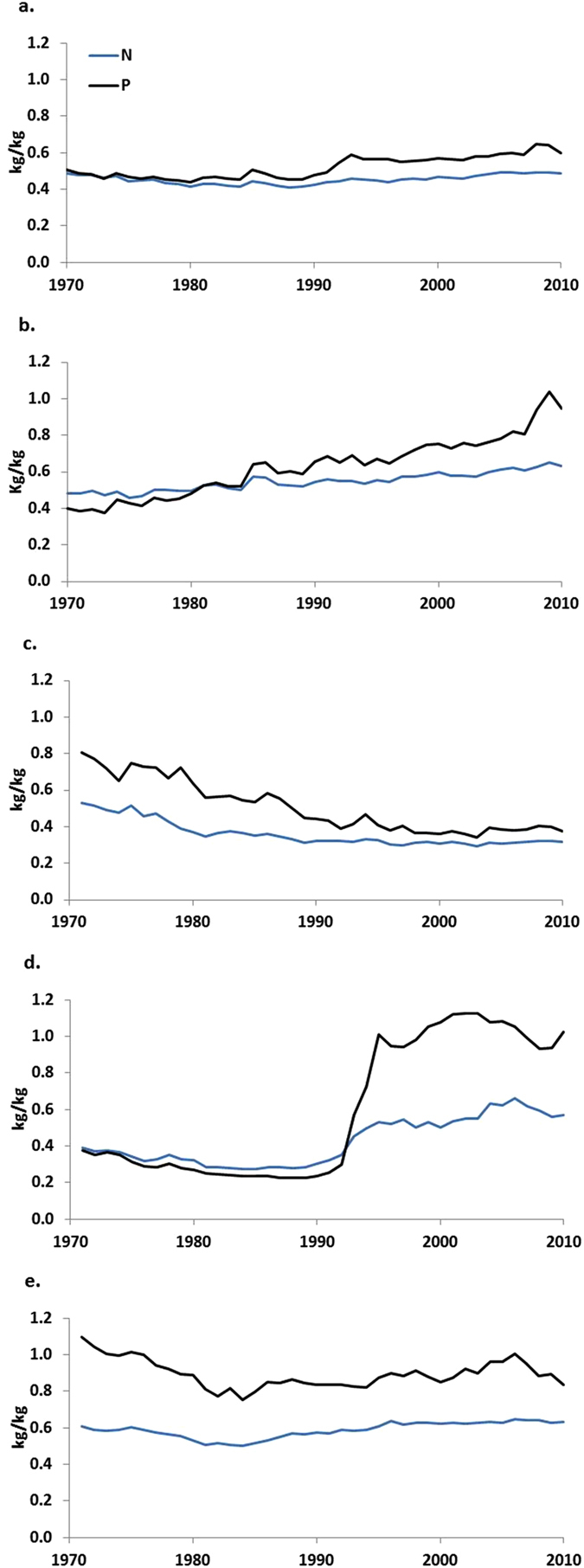
N and P use efficiency in crop production. N and P use efficiency (kg N or P in harvest per kg N or P input) for the world (**a**), high-income countries (**b**), China and India (**c**), transition countries (**d**) and sub-Saharan Africa (**e**) for the period 1970–2010. See Fig. 1 for the definition of the regions.

Global agronomic soil budgets show a nearly constant N surplus since the 1990s at a level of 40–50 kg per hectare, while the residual P budget is variable and has even decreased in recent years (Fig. 1a,b). The contribution of manure to total inputs ranges between 20 and 25% for N and between 25 and 30% for P (Figure SI1); 70–75% of P inputs come from fertilizers. The contribution from fertilizers to total N inputs is more variable (40–55%). Despite the uncertainties involved in nutrient budget estimates as discussed in the methods section, the good agreement between different global estimated budget terms is remarkable (Tables SI7–9). The global NUE has been rather constant (~0.45), while PUE shows more inter-annual variation and has increased to values ~0.6 in recent years (Fig. 2a) reflecting the fluctuations and trends in P inputs and the role of residual soil P (Fig. 1b).

Croplands in high-income countries currently have soil N surpluses that have been relatively stable since 1990, while the uptake of N continued to increase. Phosphorus surpluses have been decreasing steadily towards a small deficit in recent years, with a steady increase of crop P uptake over the whole period (Fig. 1c,d). These changes result in slowly increasing NUE and more rapidly increasing PUE to values exceeding 1 (Fig. 2b), pointing at soil P mining.

**Figure 3:**
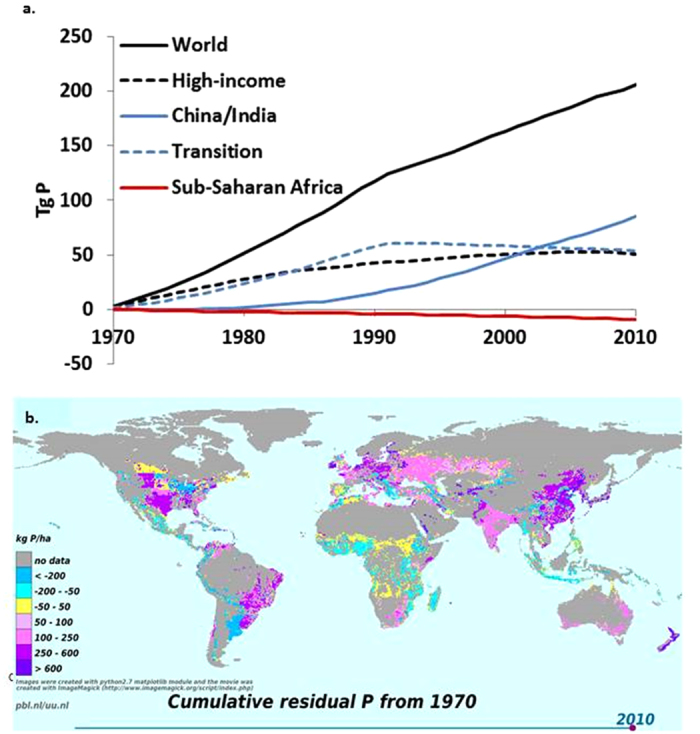
Temporal and spatial patterns of residual soil P in croplands. (**a**) Cumulative residual soil P in cropland for the world, high-income countries, China and India, transition countries and sub-Saharan Africa for the period 1970–2010. See Fig. 1 for the definition of the regions; Tg = teragram; 1 Tg = 10^12^ gram = 1 million metric ton; (**b**) Cumulative residual soil P for 0.5 by 0.5 degree grid cells for the year 2010. The SI Movie showing the changes in cumulative residual soil P during the time period 1970–2010 is available in the Supplementary Information. Figure 3b was created with python 2.7 matplotlib module^88^.

China and India have large surpluses of both nutrients (Fig. 1e,f), and are building up large soil P reserves (Fig. 3). India and China show steadily decreasing efficiencies throughout the period 1970–2010 to fairly stable values of ~0.3 for N and ~0.4 for P (Fig. 2c), which is about similar to the efficiencies in high-income countries in the 1970s.

The transition countries (Eastern Europe and former Soviet Union) have seen a major decrease of both N and P budgets since 1990 with a current agronomic N budget at a low level of 10–20 kg N per hectare, and P budgets around zero (Fig. 1g,h). The NUE reflects these changes by a sudden jump after 1990 from low values between 0.2 and 0.3, to values between 0.5 and 0.6, while PUE increased suddenly from levels close to 0.2 to more than 1.

Sub-Saharan Africa shows inputs, crop withdrawal and agronomic budgets for N at very low levels compared to other world regions (Fig. 1i). The agronomic P budget in sub-Saharan Africa fluctuates around zero, and the residual budget has small negative values (Fig. 1j). PUE fluctuates at high values (0.8–1) and NUE is lower (0.55–0.6).

In the period 1970–1980, both N and P budgets showed large surpluses in the high-income countries, but thereafter these declined drastically for P and stabilized or decreased somewhat for N. NUE and in particular PUE increased in the course of time, perhaps eventually approaching an asymptote determined by a theoretical upper limit of the NUE and PUE^15^. For both nutrients, the high-income countries follow the environmental Kuznets curves (Figure SI2)^16^,^17^. China and India are in the phase of increasing N and P surpluses and decreasing NUE and PUE, while developing countries such as in sub-Saharan Africa are still in the early phases of agricultural development with very low N and P applications and surpluses.

Turning to the contribution of biological N fixation by leguminous crops to the total N inputs, we see large differences between world regions. The global contribution of biological N fixation has been decreasing from 20% to 16% between 1970 and 2010, while in industrialized countries it has been increasing from ~17% to over 20% in the same period. China and India show a decrease from 25% to 9% in these four decades. The transition countries show an increase after 1990, but this is merely due to the sharp drop in total inputs (Fig. 1g), while production of legumes apparently did not decrease as much as total crop production. The contribution of legumes to total inputs in Sub-Saharan Africa dropped from 35% to 24% in the years 1970–2010.

### Are there nutrient imbalances?

With these dramatic changes in the evolution of nutrient dynamics over time and vast differences between world regions, we now move on to the question of nutrient imbalances. To address this question, we compare the biogeochemical behavior of N and P. Although soils can accumulate N in organic matter, such pools are bounded in size and can decompose easily, in particular due to tillage^18^. Without annual additions, such soil pools cannot supply N to crops for many years. The high N surpluses in the period 1970–1980 can be considered to be an environmental burden, since a large proportion was lost to the environment via erosion, leaching to ground- and surface water, and gaseous emissions generating severe environmental problems^19^.

In contrast, P is much less mobile in the soil system, as it is chemically absorbed by soil particles^20^. P can be lost from soil by runoff in the form of dissolved reactive P and particulate P and will leach to water systems only after (very) long excess application of P^21^,^22^. The remainder, however, of any P application to soils in any form (fertilizer, manure, waste) is retained in soil components with a continuum of bonding energies with varying degrees of reversibility, ranging from recalcitrant pools to pools containing P that is directly available to plants^20^.

The development in the high-income countries shows that the large P surpluses in the 1970s to 1980s represented a legacy for future productive capacity. The present situation in many high-income countries shows that the P fertilizer inputs and P surpluses can be reduced considerably and the PUE can be increased to high levels without a yield penalty (Fig. 1d). Our data indicate that at present some soil P mining is taking place in many high-income countries (Fig. 1d,h). This phenomenon of the legacy of soil P due to large P surpluses, such as during the 1970s and 1980s in Western Europe (Figure SI3), Eastern Europe, the Russian Federation and the USA (Movie SI2), has been pointed out earlier^1^. The data for the transition countries show that this mining can continue during prolonged periods without any apparent effect on yields (Fig. 1h). However, the inputs have been reduced to low levels and surpluses have turned into deficits, and it remains to be investigated how long this situation can persist without a yield decline by P limitation. Here, the use of a mechanistic phosphorus model can assist^1^.

The role of residual soil P in the high-income countries partly explains the increasing N:P ratio in the mineral fertilizers used, which not necessarily points to an imbalance between N and P availability for crops. Another reason is the increased use of animal manure with a high P content relative to N^23^. Nitrogen is easily lost from the livestock system mainly due to NH_3_ losses, while P is often supplied as feed supplement. This causes the N:P ratio in the manure to be much smaller than that in the feed crops supplied to livestock. Manure recycled in cropland is thus P rich and partly compensates the high N:P ratio in fertilizers applied (Figure SI4).

China and India have experienced long periods of N and P surpluses, in particular since the 1990s. Cumulative residual soil P in these two countries is now 53 and 34 Tg P for the period 1970–2010, compared to 12 Tg in Western Europe and 16 Tg in the USA (Fig. 3; SI Movie). Recent analyses indicate that in China P application rates can be reduced while increasing the P uptake by crops^5^, confirming our finding that accumulated P per hectare of crop soils in China and India exceeds the residual P in the USA, Western Europe, and the Russian Federation, revealing huge potential to reduce P application.

### Environmental implications

Agricultural^24^,^25^,^26^ and urban^27^,^28^ landscapes have a nutrient memory, i.e. the mobilized nutrients can be delivered to rivers after prolonged time periods by aquifers and sediments. In the initial phase of excessive nutrient use in agricultural systems in high-income countries in the 1970s and 1980s and at present in India and China, nutrients accumulate and landscapes function as a buffer. For the Mississippi, IMAGE-GNM calculates a storage of 83 Tg N over the 1960–2010 period in the subsoil, which is somewhat less than the estimated 142 Tg in a recent paper^25^. With declining nutrient inputs, soils may be releasing nutrients by organic matter decomposition^25^, and aquifers (particularly N)^29^ and sediments (particularly P)^30^ in lakes, reservoirs and rivers continue to deliver nutrients.

The N concentration in the Mississippi has not decreased in recent decades, despite policies to reduce nutrient loading. The flow-normalized N export by the Mississippi has even increased since 1980, and the increased N concentration at low stream flows is a strong indication that nitrate delivery by groundwater has a strong effect on river concentrations^31^.

**Figure 4:**
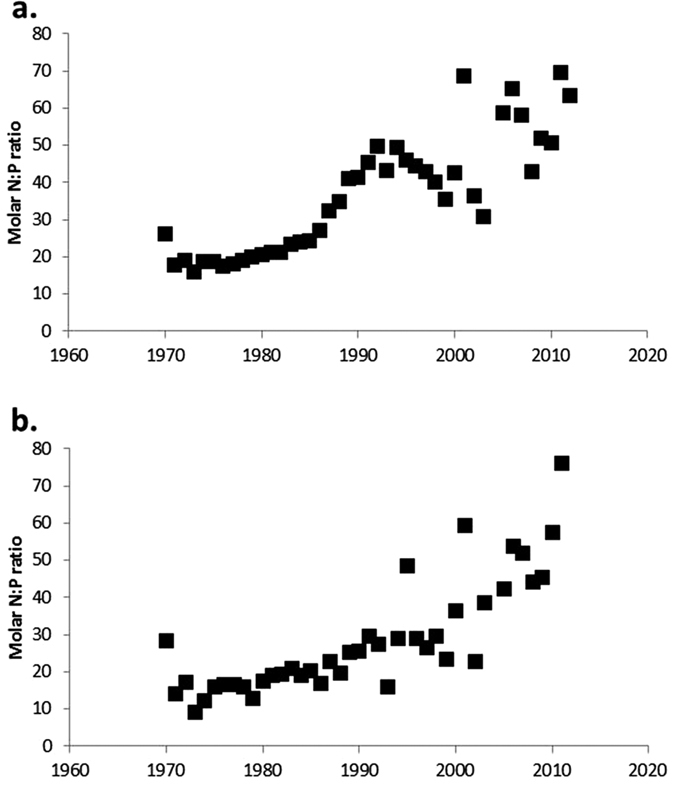
N:P molar ratio in rivers. (**a**) Rhine at Lobith and (**b**) Meuse river at station Eysden, The Netherlands. Data from the Ministry of Infrastructure and Environment^89^.

Regulations in the European Union to reduce groundwater pollution by nitrate and nutrient discharges in wastewater^32^,^33^,^34^ have not led to a reduction in N concentrations in rivers. In view of the legacy of historical large N surpluses, the EU Nitrates directive focuses on the regulation of manure N in agriculture. However, it seems to have a stronger effect on P than on N. Also increasing global prices of P fertilizer since 2005 could explain the recent decrease of P-fertilizer use. Regulations for wastewater treatment directly lead to a reduction of N and P discharge^35^. We see this phenomenon of slow or no change in N and decreasing P not only in the Rhine and Meuse (Fig. 4), but also in other European rivers draining into the English Channel, Atlantic, W. Mediterranean Sea and Northern Adriatic Sea^36^.

While increased anthropogenic nutrient loading has severe ecological impacts, the situation of nutrient reduction causing changing nutrient ratios described above may have additional impacts. When N and P are discharged to surface water in excess over silicon (Si) with respect to the requirements of siliceous algae (diatoms), often undesirable non/diatom algal species will develop. Many phytoplankton species causing harmful algal blooms have physiological adaptive strategies that favour them under conditions of elevated N:P conditions^37^,^38^, especially when silicon (Si) flows are not changing or declining^39^. The construction of dams and development of reservoirs has led to declining Si transport by rivers^40^, and the complexity is even exacerbated by impacts of several conspiring global change processes on the biogeochemical transformations in river basins, such as climate change, land use change, urbanization and consumptive water use.

### Lessons learnt

China and India are now in the phase of increasing N and P surpluses and decreasing nutrient use efficiencies, but will eventually start to increase their efficiency and reduce nutrient surpluses, and at the same time construct sewage systems with wastewater treatment facilities. By doing so, they will likely face similar unintentional side effects associated with landscape legacies as we now experience in e.g. Europe e.g. ref. 41.

Many developing countries such as those in sub-Saharan Africa are still in the early phases of agricultural development with minimal N and P application rates and surpluses, and often soil N and P resources are being mined (Figs 1i,j and 3). For securing food supply for the growing populations, farmers must substantially increase the supply^42^,^43^ but also improve the management of both N and P^44^. Strongly weathered tropical soils with strong chemical P retention are widespread in many developing countries^45^ and high P inputs are needed to arrive at a similar P availability as currently seen in high-income countries^46^. What is thus needed is an efficient nutrient management that avoids large N surpluses and aquifer pollution with nitrate, and that accounts for the effect of residual soil P.

## Concluding Remarks

With regard to N, developing countries may benefit from the management and technology that are now becoming common in high-income countries and that aim at reducing environmental N losses and target high N use efficiencies simultaneously; ideally they can move directly to a system with high yields *and* high N use efficiency^15^ to avoid N loading of aquifers and gaseous emissions of nitrous oxide, nitric oxide and ammonia. Integrated crop, soil and nutrient management focusing on entire crop rotations rather than single crops helps to achieve high N use efficiencies in crop production. Legumes are important in such rotations to bring in additional N (e.g. in sub-Saharan Africa) or to substitute fertilizer N (e.g. in Europe). However, an important policy and management issue is that for legumes to play an important role, the P availability needs to be increased, and problems associated with soil acidity have to be solved, since the nitrogen fixation process is particularly thwarted under low P and acid soil conditions^47^. As to combining high nutrient use efficiency and low emissions, the use of simple indicators such as N and P budgets should be advocated^48^.

We note that for improving P use efficiencies, a long term farming system perspective is needed. The availability of P for crop uptake and P use efficiency can be improved by an adequate and gradual build-up of residual soil P, while accounting for risks for aquatic ecology by minimizing runoff losses. Subsidizing the use of synthetic P fertilizer is a possible measure to enhance this P build up, since during this accumulation phase of residual soil P, the PUE will be relatively low.

Efficient use of fertilizers requires a combination of good agronomic practices with efficient nutrient management using a mix of organic and mineral sources^49^,^50^. Such agronomic practices include the use of high-yielding crop varieties that are adapted to local climate and soil conditions, recycling and application of organic matter such as crop residues and animal manure, soil fertility management such as liming to improve P availability in strongly weathered soils, and appropriate protection of crops against weeds, pests and diseases. Where possible, irrigation may also help to improve nutrient use efficiency. Policies for good soil fertility and agronomic management must be devised that also consider climate goals to reduce emissions of nitrous oxide and increase carbon storage^51^,^52^. To achieve all this, governments, extension services, international organizations and private sector partners need to build capacity in nutrient management designed to match local conditions to support and sustain crop productivity. There seems to be an increasing awareness that current national and international future targets for research and development budgets should increase, particularly in Africa^53^,^54^,^55^.

## Methods

### General

The data presented in this study are from the database used in the IMAGE-Global Nutrient Model (GNM)^11^,^12^ to compute soil nitrogen (N) and phosphorus (P) budgets. IMAGE-GNM is part of the Integrated Model to Assess the Global Environment (IMAGE)^56^. The data are spatially explicit (see for example SI movie). The most complete descriptions of the procedure to distribute the various budget terms is provided by Beusen *et al*.^13^ and Bouwman^57^, the latter publication has supporting information with the executable of the distribution model, documentation and manual, and input files for running the software. The annual agronomic soil nutrient budget includes the N and P inputs and outputs. N inputs comprise biological N fixation (*N*_fix_), atmospheric N deposition (*N*_dep_), application of synthetic N fertilizer (*N*_fert_) and animal manure (*N*_man_). Outputs in the soil N budget are N withdrawal from the field through crop harvesting (*N*_withdr_). The agronomic soil N budget (*N*_budget_) is calculated as follows:


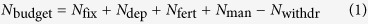


A positive budget implies a surplus, and a negative budget is a deficit. For P the same approach was used to compute the agronomic soil budget, P inputs being animal manure and fertilizer:


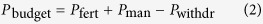


Residual soil P also account for runoff losses, and represents the accumulation or depletion of phosphorus in soil landscapes over multiple seasons.


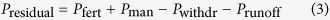


Nutrient inputs and outputs for all crops cultivated in cropland systems are aggregated, including leguminous crops that fix atmospheric N to meet part of their N requirement. Legumes are included as they often are part of a rotation with cereals, their fixed N is part of the rotation’s N budget, and their P requirement is relatively large^58^.

Data on crop production, livestock, and fertilizer use are from FAOSTAT^9^,^10^. For calculating the soil N and P budgets, statistical data are used for all countries of the world from FAOSTAT^9^,^10^, and subnational data for USA^59^, China^60^,^61^,^62^, and Europe^63^. For countries where subnational data are used, the data is scaled so that the national total matches the FAOSTAT data.

FAOSTAT data cover the period 1961 till the most recent year. USDA data^59^ cover a longer period; the start year of Chinese data^60^,^61^,^62^ is variable. For all data sources the rule is that if available, the time series 1961 or the earliest year after 1961 to the most recent year is used; items having years with no data are linearly interpolated. The distribution of subnational data for the first available year is used together with FAO data for the whole country for preceding years. If data for similar categories are available, the linear trend for that item is used to compute preceding years for the item with missing data. In the results presented in this study, the first year considered in 1970, which is regarded less uncertain than data years prior to 1970, and this is consistent with the first year considered by the IMAGE model^56^.

Subsequently, we discuss the data on crop production and nutrient uptake, crop residues, fertilizer use by crop, animal manure, biological N fixation, deposition and runoff losses.

### Crop production and nutrient uptake

Crops are first grouped according to the list of 34 crops distinguished by FAO Agriculture Towards 2030 and 2050 studies^64^,^65^ (Table SI2). Using estimated N and P contents for the 34 crop groups (Table SI2), the amounts of nutrients in the harvested parts are computed for each country, state (USA) or province (China). This information is used to distribute information generated by IMAGE at the scale of world regions to the country scale. This procedure warrants consistency between IMAGE output and GNM calculations at the country and grid scale.

For spatial distribution of N inputs, outputs and budgets on 0.5 by 0.5 degree or 5 by 5 minute resolution, the 34 crops were grouped to 3 groups, i.e. legumes, wetland rice, and upland crops (see Table SI2). The inputs and outputs of legumes, wetland rice, upland crops are finally aggregated.

Fodder crop production is retrieved from FAOSTAT^9^ (Table SI3). Since data on fodder crop production are not available for the states of the USA and provinces of China, the distribution of fodder crop production from FAOSTAT for China, USA over the states/provinces is mimicked by using the distribution of cattle, dairy and pigs. Splitting the country data for France, Italy and Spain into regions is done in the same way.

Fodder products are split in two classes, i.e. (i) fodder for nondairy and dairy cattle (cattle) (ii) fodder for nondairy, dairy cattle and pigs (all) (Table SI3). The nutrients in harvested fodder crops including the leguminous fodders are added to that of the crop group of upland crops.

The nutrient export and input in seeds has been neglected, assuming that the seed used is from the same grid cell, so seed harvest and seeding is budget neutral.

### Crop residues

The estimated N and P contents in crop residues listed in Table SI4 have been used to estimate the N loss during burning (P has no gas phase and is thus returned to the soil in the ash) and the withdrawal from the plant-soil system by use of residues as feed for livestock (Table SI5).

Data on burning of crop residues for world regions are from the IMAGE model^56^, while the data of feed use of crop residues are from Herrero *et al*.^66^.

### Fertilizer use by crop

The most recent inventories of fertilizer use by crop (http://www.fertilizer.org//En/Statistics/Agriculture_Committee_Databases.aspx) cover only 23 countries and lack data on fertilizer use in grassland, while Fertilizer Use By Crop (FUBC) edition 5^67^ includes a much larger number of countries and specific information of fertilizer use in grasslands. In FUBC5 the total N and P fertilizer use is provided per country and per crop or grass with the area where the fertilizer is applied. This information is used to calculate an average application rate (kg/ha) per country for the three crop groups (upland crops, legumes and rice). This is used as the application rate for the year 2000. Countries with no data are assigned a regional average (in this case IMAGE region, Table SI1) application rate per crop.

The next step is to obtain the crop area from IMAGE, USDA and Chinese data for all crops and aggregate this for each country for the 3 crop groups. The N or P crop yield, calculated as the N or P uptake (based on FAOSTAT) divided by the harvested area, is used to scale the distribution of fertilizers in any other year before or after 2000. More details on this procedure can be found in Beusen *et al*.^12^.

### Animal manure

Country-scale data on animal stocks are from the FAOSTAT data^9^,^10^, and subnational data for China^60^,^61^,^62^ and the USA^59^. Chinese statistics do not provide animal stocks for all years. Therefore, the first or last year with data on animal stocks is used for each animal category using the trend in the stocks of the other animal categories. The USA statistics provide data for five classes (dairy cattle, beef cattle, pigs, poultry and sheep and goats). We use the sum of stocks of cattle and sheep and goats as a proxy to fill in the stocks for the other animal classes (like horses, camels, mules, asses etc.).

Animal manure production is calculated by multiplying animal stocks and excretion rates (Table SI6). This approach with constant excretion rates in time thus results in changing excretion per unit of product when the animal productivity changes (milk production per cow or carcass weight). The input of manure for the soil budget of cropland (equation 1) is total manure, excluding (i) manure excreted in the meadow during grazing, (ii) manure excreted outside the agricultural system (for example in urban areas, forests and along roadsides or manure collected in lagoons^68^) and (iii) manure used as fuel or for other purposes^69^. Animal manure available for application includes thus all stored or collected manure in animal houses or other systems (e.g. kraal). This amount is corrected for 20% NH_3_-N volatilization from such storage systems^70^. More details on this approach are provided by Bouwman *et al*.^57^.

We assumed that in most high-income countries, 50% of the available animal manure from animal houses and storage systems is applied to cropland and the remainder to grassland^71^. In most developing countries, 95% of the available manure is assumed to be applied to cropland and 5% to grassland, thus accounting for stubble grazing and manure excretion in croplands, and the lower economic importance of grass compared to crops in developing countries^72^. For EU countries we used maximum manure application rates of 170–250 kg N ha^−1^ yr^−1^ based on existing regulations. The availability of manure for spreading in croplands varies in the IMAGE model by changes in the distribution of animals between pastoral and mixed systems, and changes in the amount of time that ruminants are in the meadow versus the stable; the latter is governed by the amount of grass in the feed rations of ruminants.

### Biological N fixation

Data on biological N fixation by leguminous crops (pulses, groundnuts and soybeans) are obtained from the N in the harvested product. The approach assumes a harvest index (HI, ratio seed grains: aboveground biomass) of 0.5, and a ratio roots: aboveground biomass of 0.19. With these parameters the biomass in grains, straw and roots can be calculated. The total N in the plants can be calculated with the production data and the N content of legume seeds (3.5% for all crops except for soybean where 6.2% is used), straw (0.8%) and roots (0.8%). The N fixation in kg per hectare soybean according to Salvagiotti *et al*.^73^ is 0.66*(kg plant-N/ha)-19. This yields N fixation estimates that amount to 0.6 times total plant N. Hence we simplified the approach by using this 0.6 coefficient to all pulses. With this approach, any change in the rate of biological N fixation by legumes is the result of yield changes for pulses and soybeans.

Some fodder crops (clover, alfalfa an legumes for silage) are legumes, but these are grouped with upland crops. In contrast to the other legumes, N fixation is assumed to be equal to the N fodder production (production times the N content). For non-symbiotic biological N fixation for non-leguminous crops (5 kg ha^−1^ yr^−1^) and wetland rice (25 kg ha^−1^yr^−1^) estimates of Smil^74^ are used.

### Atmospheric deposition

Atmospheric N deposition rates are obtained by scaling the deposition fields for 2000^75^ using emission inventories for N gases for the corresponding years from the IMAGE model^56^. Atmospheric deposition of P is a minor flux globally^76^ and has been neglected.

### Runoff loss

IMAGE-GNM distinguishes three runoff nutrient pathways, i.e. (i) losses from recent nutrient applications in the form of fertilizer, manure or organic matter^77^, (ii) a “memory” effect related to long-term historical changes in soil nutrient inventories^78^,^79^, and (iii) P loss by weathering and transport by subsurface runoff and aquifers. The approach uses erosion soil loss estimates from Cerdan *et al*.^80^, who used measurement data to develop a model based on slope, soil texture and land cover type to estimate country aggregated soil-loss rates for cropland, grassland and natural vegetation. The initial P stock (year 1900) in the top 30 cm is taken from a recent inventory^81^. Inputs and outputs of the soil budget are assumed to occur in the top 30 cm; the model replaces P lost by erosion with fresh soil material (with the initial soil P content) at the bottom. The model yields high values of P runoff loss as a fraction of P inputs for regions with low P inputs due to the relative important contribution of P in soil material, such as in the Russian Federation and sub-Saharan Africa. In other regions, P losses by runoff range between 10 and 30% of P inputs. For N the soil organic C content, which is assumed to be constant over time, is used as a basis to calculate N in eroded soil material using land-use specific C:N ratios (soil C:N for arable land 12, for grassland 14 and for soils under natural vegetation 14). In the year 2010, this approach yields runoff N losses of 4–16% of total inputs and 10–40% of total N losses, depending on the combination of soil properties and slope, and climatic conditions determining denitrification and leaching losses. Details on the IMAGE-GNM approach to compute erosion losses of N and P are provided by Beusen *et al*.^11^.

The calculation of P loss from landscapes by weathering and transport by subsurface flow and aquifers to surface water is based on a recent study^82^ as outlined in detail in the IMAGE-GNM description^11^. In short, this approach uses a background concentration in the runoff via aquifers specified for each lithological class^83^, water runoff, furthermore a correction factor for soil shielding, and local mean annual air temperature using an Arrhenius equation.

### Uncertainties in the nutrient budgets

We recognize that there are many uncertainties involved in our budget calculations. We slightly underestimate the withdrawal of nutrients from fields by ignoring burning of crop residues (2.7 Tg N in 2010, mostly in developing countries)^84^ and the use of straw as animal feed (5.1 Tg N and 0.6 Tg P in 2010, mostly in Southern Asia and sub-Saharan Africa) (see Table SI5). Moreover, inputs may be underestimated by neglecting the use of human excreta to fertilize cropland, such as in China in the 1970s^85^. Global manure inputs in croplands of circa 35 Tg N yr^−1^ in 2010 may vary by ±30%^13^, and regarding fertilizer consumption the statistics have large but unknown uncertainties. For example, there is a discrepancy between the different statistics, with 95 Tg N yr^−1^ from FAO^86^ and 104 Tg N yr^−1^ from IFA^87^ for global N fertilizer use and large differences for China (23.7 Tg N in 2010 from FAO, versus 32.6 Tg N from IFA). Uptake estimates are based on constant N and P contents and crop production data from FAO^86^ with unknown uncertainties. Some minor nutrient budget terms are neglected. P loss by leaching, nutrient inputs in seeds are neglected, and wind erosion losses are ignored. However, using the time series in a consistent way, the trends in the crop production system are probably depicted with less uncertainty than the absolute levels.

## Additional Information

**How to cite this article**: Bouwman, A. F. *et al*. Lessons from temporal and spatial patterns in global use of N and P fertilizer on cropland. *Sci. Rep.* **7**, 40366; doi: 10.1038/srep40366 (2017).

**Publisher's note:** Springer Nature remains neutral with regard to jurisdictional claims in published maps and institutional affiliations.

## Supplementary Material

Supplementary Information

Supplementary Movie

Supplementary Dataset 1

Supplementary Dataset 2

Supplementary Dataset 3

Supplementary Dataset 4

Supplementary Dataset 5

Supplementary Dataset 6

Supplementary Dataset 7

Supplementary Dataset 8

Supplementary Dataset 9

Supplementary Dataset 10

Supplementary Dataset 11

Supplementary Dataset 12

Supplementary Dataset 13

Supplementary Dataset 14

Supplementary Dataset 15

Supplementary Dataset 16
