# Supplementary material for: Lessons from temporal and spatial patterns in global use of N and P fertilizer on cropland
Source: Sci Rep. 2017 Jan 13;7:40366. doi: 10.1038/srep40366 (PMC5234009; doi:10.1038/srep40366)
Supplement: Supplementary Information [file srep40366-s1.pdf]

Lessons from temporal and spatial patterns in global use of N and P fertilizer on cropland

Supporting information (SI)

A.F. Bouwman[1,2], A.H.W. Beusen[1,2], L. Lassaletta[2], D. Van Apeldoorn[3], H. van Grinsven[2], J. Zhang[1,4], M.K. van Ittersum[5]

[1] Department of Earth Sciences - Geochemistry, Faculty of Geosciences, Utrecht University, P.O. Box 80021, 3508 TA Utrecht, The Netherlands.
[2]PBL Netherlands Environmental Assessment Agency, PO Box 303, 3720 AH Bilthoven, The Netherlands.
[3] Farming Systems Ecology group, Wageningen University, PO Box 430, 6700 AK Wageningen, The Netherlands
[4] Center for Earth System Science, Tsinghua University, 100084 Beijing, China
[5] Plant Production Systems Group, Wageningen University, P.O. Box 430, 6700 AK Wageningen, The Netherlands

Contents

SI Tables
Table SI1. Region definition
Table SI2. List of the 34 crops distinguished by FAO Agriculture Towards 2030 and 2050 studies [35,36], their allocation to the 3 crop groups of IMAGE-GNM and their N, P and dry matter content.
Table SI3. List of fodder crops[a] included in the data.
Table SI4. N and P as a fraction of dry matter of crop residues for 8 crop groups.
Table SI5. Crop residues used as animal feed in different world regions, and amount of N and P withdrawal from the soil-plant system around 2010.
Table SI6. Excretion rates for the different animal categories
Table SI7. Comparison of global N budget terms from different sources for the year 1995.
Table SI8. Comparison of global N budget terms from different sources for the year 2009.
Table SI9. Comparison of global P budget terms from different sources for the year 2000 and 2013 for croplands.

SI Movie
Click the file (Global cumulative residual phosphorus 1970-2010. GIF) to see the movie. SI_Images were created with python 2.7 matplotlib module [1] and the movie with ImageMagick (http://www.ImageMagick.org/script/index.php).

SI Figures
Figure SI1. Global balance terms and budget/residue for N (top) and P (bottom). Data for regional N and P soil budget terms are in the SI_datafiles.

Figure SI2. Environmental Kutznets curve showing stylized relationship between income level and N surplus (N_{sur}). Source: Zhang et al. [2].

Figure SI3. Relationship between N inputs and N uptake (top panel) and P inputs and P uptake (bottom panel) by crops in Western Europe. The hysteresis of N inputs can be explained by increased N efficiencies, while P is because of the accumulation of residual P.

Figure SI4. N:P ratio (weight basis) of a) new N inputs (N fertilizer, N fixation) and new P inputs (P fertilizer); and of b) total N and P inputs.

Figure SI5a. Global 0.5 by 0.5 degree distribution of manure P inputs for 2010.
Figure SI5b. Global 0.5 by 0.5 degree distribution of synthetic fertilizer P inputs for 2010.
Figure SI5c. Global 0.5 by 0.5 degree distribution of surface runoff P loss for 2010.
Figure SI5d. Global 0.5 by 0.5 degree distribution of weathering P losses from soil landscapes for 2010.
Figure SI5e. Global 0.5 by 0.5 degree distribution of crop P uptake for 2010.

SI Datafiles

N and P input and output data by the 26 world regions (for region definition see Table SI1) and the world (region 27) (SI_Datafiles.zip file)**.**

Region definitions are in Table SI1. There are two files with cropland area (Ncropland_area and P_cropland area). The other files provide the regional data for the different N input and output terms in the soil nitrogen (N) and phosphorus (P) budgets for croplands: atmospheric deposition (for N only), biological N fixation, fertilizer use and manure application for N and P, runoff losses and crop uptake (for N and P), and P weathering. Finally, N and P use efficiency is provided in two files. Below is the list of files, with their contents and unit.

Ncropland_area.csv	Cropland area (km^2)
Ncropland_deposition.csv`	Atmospheric N deposition (kg N per year)
Ncropland_fertilizer.csv	N fertilizer application (kg N per year)
Ncropland_fixation.csv	Biological N fixation (kg N per year)
Ncropland_manure.csv	N manure application (kg N per year)
Ncropland_runoff.csv	N runoff (kg N per year)
Ncropland_uptake.csv	Crop N uptake (kg N per year)
Ncropland_use_eff	N use efficiency, i.e. crop uptake / total inputs (-)
Pcropland_area.csv	Cropland área (km^2)
Pcropland_fertilizer.csv	P fertilizer application (kg P per year)
Pcropland_manure.csv	P manure application (kg P per year)
Pcropland_runoff.csv	P runoff (kg P per year)
Pcropland_weathering.csv	P runoff from weathering (kg P per year)
Pcropland_uptake.csv	Crop uptake (kg P per year)
Pcropland_use_eff	P use efficiency, i.e. crop uptake / total inputs (-)
Pcropland_residue.csv	Residual soil P 1970-2010 (kg P per year), calculated as fertilizer+manure-uptake-runoff-weathering

SI Literature

Table SI1. Region definition.

The definition of the regions used in the IMAGE model [3] is provided in the table below. In the main text these regions are further aggregated to Industrialized countries, China and India, transition countries and developing countries.). Industrialized countries comprise Canada (region 1), USA (2), Western Europe (region 11), Japan (23), Oceania (24), China and India region includes the China region (20) and India (18), transition countries (Eastern Europe, 12, and former Soviet Union, 12, 14, 15, 16) and sub-Saharan Africa (Africa excluding Algeria, Egypt, Libya, Morocco, Tunisia, Western Sahara and South Africa).
Region number, country name, ISO country code, and region name..

Region #	Country name	ISO	Region name
1	CANADA	124	Canada
2	SAINT PIERRE AND MIQUELON	666	USA
2	UNITED STATES	840	USA
2	UNITED STATES MINOR OUTLYING ISLANDS	581	USA
3	MEXICO	484	Mexico
4	ANGUILLA	660	Rest Central America
4	ANTIGUA AND BARBUDA	28	Rest Central America
4	ARUBA	533	Rest Central America
4	BAHAMAS	44	Rest Central America
4	BARBADOS	52	Rest Central America
4	BELIZE	84	Rest Central America
4	BERMUDA	60	Rest Central America
4	CAYMAN ISLANDS	136	Rest Central America
4	COSTA RICA	188	Rest Central America
4	CUBA	192	Rest Central America
4	DOMINICA	212	Rest Central America
4	DOMINICAN REPUBLIC	214	Rest Central America
4	EL SALVADOR	222	Rest Central America
4	GRENADA	308	Rest Central America
4	GUADELOUPE	312	Rest Central America
4	GUATEMALA	320	Rest Central America
4	HAITI	332	Rest Central America
4	HONDURAS	340	Rest Central America
4	JAMAICA	388	Rest Central America
4	MARTINIQUE	474	Rest Central America
4	MONTSERRAT	500	Rest Central America
4	NETHERLANDS ANTILLES	530	Rest Central America
4	NICARAGUA	558	Rest Central America
4	PANAMA	591	Rest Central America
4	PUERTO RICO	630	Rest Central America
4	SAINT KITTS AND NEVIS	659	Rest Central America
4	SAINT LUCIA	662	Rest Central America
4	SAINT VINCENT AND THE GRENADINES	670	Rest Central America
4	TRINIDAD AND TOBAGO	780	Rest Central America
4	TURKS AND CAICOS ISLANDS	796	Rest Central America
4	VIRGIN ISLANDS_BRITISH	92	Rest Central America
4	VIRGIN ISLANDS_U.S.	850	Rest Central America
5	BRAZIL	76	Brazil
6	ARGENTINA	32	Rest South America
6	BOLIVIA	68	Rest South America

6	BOUVET ISLAND	74	Rest South America
6	CHILE	152	Rest South America
6	COLOMBIA	170	Rest South America
6	ECUADOR	218	Rest South America
6	FALKLANDS ISLANDS (MALVINAS)	238	Rest South America
6	FRENCH GUIANA	254	Rest South America
6	GUYANA	328	Rest South America
6	PARAGUAY	600	Rest South America
6	PERU	604	Rest South America
6	SOUTH GEORGIA AND THE SOUTH SANDWICH ISLANDS	239	Rest South America
6	SURINAME	740	Rest South America
6	URUGUAY	858	Rest South America
6	VENEZUELA	862	Rest South America
7	ALGERIA	12	NorthernAfrica
7	EGYPT	818	NorthernAfrica
7	LIBYAN ARAB JAMAHIRIYA	434	NorthernAfrica
7	MOROCCO	504	NorthernAfrica
7	TUNISIA	788	NorthernAfrica
7	WESTERN SAHARA	732	NorthernAfrica
8	BENIN	204	Western Africa
8	BURKINA FASO	854	Western Africa
8	CAMEROON	120	Western Africa
8	CAPE VERDE	132	Western Africa
8	CENTRAL AFRICAN REPUBLIC	140	Western Africa
8	CHAD	148	Western Africa
8	CONGO	178	Western Africa
8	CONGO_THE DEMOCRATIC REPUBLIC OF THE	180	Western Africa
8	CÔTE D'IVOIRE	384	Western Africa
8	EQUATORIAL GUINEA	226	Western Africa
8	GABON	266	Western Africa
8	GAMBIA	270	Western Africa
8	GHANA	288	Western Africa
8	GUINEA	324	Western Africa
8	GUINEA-BISSAU	624	Western Africa
8	LIBERIA	430	Western Africa
8	MALI	466	Western Africa
8	MAURITANIA	478	Western Africa
8	NIGER	562	Western Africa
8	NIGERIA	566	Western Africa
8	SAINT HELENA	654	Western Africa
8	SAO TOME AND PRINCIPE	678	Western Africa
8	SENEGAL	686	Western Africa
8	SIERRA LEONE	694	Western Africa
8	TOGO	768	Western Africa
9	BURUNDI	108	EasternAfrica
9	COMOROS	174	EasternAfrica
9	DJIBOUTI	262	EasternAfrica
9	ERITREA	232	EasternAfrica
9	ETHIOPIA	231	EasternAfrica
9	KENYA	404	EasternAfrica
9	MADAGASCAR	450	EasternAfrica

9	MAURITIUS	480	EasternAfrica
9	MAYOTTE	175	EasternAfrica
9	RÉUNION	638	EasternAfrica
9	RWANDA	646	EasternAfrica
9	SEYCHELLES	690	EasternAfrica
9	SOMALIA	706	EasternAfrica
9	SUDAN	736	EasternAfrica
9	UGANDA	800	EasternAfrica
10	SOUTH AFRICA	710	Southern Africa
11	ANDORRA	20	Western Europe
11	AUSTRIA	40	Western Europe
11	BELGIUM	56	Western Europe
11	DENMARK	208	Western Europe
11	FAROE ISLANDS	234	Western Europe
11	FINLAND	246	Western Europe
11	FRANCE	250	Western Europe
11	GERMANY	276	Western Europe
11	GIBRALTAR	292	Western Europe
11	GREECE	300	Western Europe
11	HOLY SEE (VATICAN CITY STATE)	336	Western Europe
11	ICELAND	352	Western Europe
11	IRELAND	372	Western Europe
11	ITALY	380	Western Europe
11	LIECHTENSTEIN	438	Western Europe
11	LUXEMBOURG	442	Western Europe
11	MONACO	492	Western Europe
11	NETHERLANDS	528	Western Europe
11	NORWAY	578	Western Europe
11	PORTUGAL	620	Western Europe
11	SAN MARINO	674	Western Europe
11	SPAIN	724	Western Europe
11	SVALBARD AND JAN MAYEN	744	Western Europe
11	SWEDEN	752	Western Europe
11	SWITZERLAND	756	Western Europe
11	UNITED KINGDOM	826	Western Europe
12	ALBANIA	8	Central Europe
12	BOSNIA AND HERZEGOVINA	70	Central Europe
12	BULGARIA	100	Central Europe
12	CROATIA	191	Central Europe
12	CYPRUS	196	Central Europe
12	CZECH REPUBLIC	203	Central Europe
12	ESTONIA	233	Central Europe
12	HUNGARY	348	Central Europe
12	LATVIA	428	Central Europe
12	LITHUANIA	440	Central Europe
12	MACEDONIA_THE FORMER YUGOSLAV REPUBLIC OF	807	Central Europe
12	MALTA	470	Central Europe
12	POLAND	616	Central Europe
12	ROMANIA	642	Central Europe
12	SLOVAKIA	703	Central Europe
12	SLOVENIA	705	Central Europe

12	YUGOSLAVIA	891	Central Europe
13	TURKEY	792	Turkey
14	BELARUS	112	Ukraine +
14	MOLDOVA_REPUBLIC OF	498	Ukraine +
14	UKRAINE	804	Ukraine +
15	KAZAKSTAN	398	Asia-Stan
15	KYRGYZSTAN	417	Asia-Stan
15	TAJIKISTAN	762	Asia-Stan
15	TURKMENISTAN	795	Asia-Stan
15	UZBEKISTAN	860	Asia-Stan
16	ARMENIA	51	Russia +
16	AZERBAIJAN	31	Russia +
16	GEORGIA	268	Russia +
16	RUSSIAN FEDERATION	643	Russia +
17	BAHRAIN	48	Middle East
17	IRAN_ISLAMIC REPUBLIC OF	364	Middle East
17	IRAQ	368	Middle East
17	ISRAEL	376	Middle East
17	JORDAN	400	Middle East
17	KUWAIT	414	Middle East
17	LEBANON	422	Middle East
17	OMAN	512	Middle East
17	QATAR	634	Middle East
17	SAUDI ARABIA	682	Middle East
17	SYRIAN ARAB REPUBLIC	760	Middle East
17	UNITED ARAB EMIRATES	784	Middle East
17	YEMEN	887	Middle East
18	INDIA	356	India
19	KOREA_DEMOCRATIC PEOPLE'S REPUBLIC OF	408	Korea
19	KOREA_REPUBLIC OF	410	Korea
20	CHINA	156	China +
20	HONG KONG	344	China +
20	MACAU	446	China +
20	MONGOLIA	496	China +
20	TAIWAN_PROVINCE OF CHINA	158	China +
21	BRUNEI DARUSSALAM	96	SoutheasternAsia
21	CAMBODIA	116	SoutheasternAsia
21	LAO PEOPLE'S DEMOCRATIC REPUBLIC	418	SoutheasternAsia
21	MALAYSIA	458	SoutheasternAsia
21	MYANMAR	104	SoutheasternAsia
21	PHILIPPINES	608	SoutheasternAsia
21	SINGAPORE	702	SoutheasternAsia
21	THAILAND	764	SoutheasternAsia
21	VIET NAM	704	SoutheasternAsia

22	EAST TIMOR	626	Indonesia +
22	INDONESIA	360	Indonesia +
22	PAPUA NEW GUINEA	598	Indonesia +
23	JAPAN	392	Japan
24	AMERICAN SAMOA	16	Oceania
24	AUSTRALIA	36	Oceania
24	CHRISTMAS ISLAND	162	Oceania
24	COCOS (KEELING) ISLANDS	166	Oceania
24	COOK ISLANDS	184	Oceania
24	FIJI	242	Oceania
24	FRENCH POLYNESIA	258	Oceania
24	FRENCH SOUTHERN TERRITORIES	260	Oceania
24	GUAM	316	Oceania
24	HEARD ISLAND AND MCDONALD ISLANDS	334	Oceania
24	KIRIBATI	296	Oceania
24	MARSHALL ISLANDS	584	Oceania
24	MICRONESIA_FEDERATED STATES OF	583	Oceania
24	NAURU	520	Oceania
24	NEW CALEDONIA	540	Oceania
24	NEW ZEALAND	554	Oceania
24	NIUE	570	Oceania
24	NORFOLK ISLAND	574	Oceania
24	NORTHERN MARIANA ISLANDS	580	Oceania
24	PALAU	585	Oceania
24	PITCAIRN	612	Oceania
24	SAMOA	882	Oceania
24	SOLOMON ISLANDS	90	Oceania
24	TOKELAU	772	Oceania
24	TONGA	776	Oceania
24	TUVALU	798	Oceania
24	VANUATU	548	Oceania
24	WALLIS AND FUTUNA	876	Oceania
25	AFGANISTAN	4	Rest Southern Asia
25	BANGLADESH	50	Rest Southern Asia
25	BHUTAN	64	Rest Southern Asia
25	BRITISH INDIAN OCEAN TERRITORY	86	Rest Southern Asia
25	MALDIVES	462	Rest Southern Asia
25	NEPAL	524	Rest Southern Asia
25	PAKISTAN	586	Rest Southern Asia
25	SRI LANKA	144	Rest Southern Asia
26	ANGOLA	24	Rest Southern Africa
26	BOTSWANA	72	Rest Southern Africa
26	LESOTHO	426	Rest Southern Africa
26	MALAWI	454	Rest Southern Africa
26	MOZAMBIQUE	508	Rest Southern Africa
26	NAMIBIA	516	Rest Southern Africa
26	SWAZILAND	748	Rest Southern Africa
26	TANZANIA_UNITED REPUBLIC OF	834	Rest Southern Africa
26	ZAMBIA	894	Rest Southern Africa
26	ZIMBABWE	716	Rest Southern Africa

Greenland and Antarctica are not included in the datafiles

GREENLAND	304	Greenland
ANTARCTICA	10	Antarctica

Table SI2. List of the 34 crops distinguished by FAO Agriculture Towards 2030 and 2050 studies [35,36], their allocation to the 3 crop groups of IMAGE-GNM and their N and P content on dry matter basis, and dry matter content of the harvested product.

Crops	Name	Crop groups[a]	N content (-)	P content (-)	Dry matter (-)
1	Wheat	1 (1)	0.019	0.0034	0.880
2	Rice	3 (2)	0.013	0.0025	0.880
3	Maize	1 (3)	0.014	0.0029	0.880
4	Barley	1 (1)	0.017	0.0036	0.880
5	Millet	1 (4)	0.015	0.0030	0.850
6	Sorghum	1 (4)	0.015	0.0030	0.850
7	Other cereals	1 (1)	0.016	0.0031	0.870
8	Potato	1 (6)	0.003	0.0005	0.212
9	Sweet potato	1 (6)	0.003	0.0003	0.272
10	Cassava	1 (6)	0.002	0.0004	0.326
11	Other root crops	1 (6)	0.003	0.0004	0.270
12	Plantain	1 (8)	0.002	0.0003	0.653
13	Sugar beet	1 (8)	0.002	0.0004	0.260
14	Sugar cane	1 (8)	0.002	0.0004	0.232
15	Pulses	2 (5)	0.035	0.0025	0.850
16	Vegetables	1 (8)	0.002	0.0003	0.070
17	Banana	1 (8)	0.002	0.0002	0.257
18	Citrus	1 (8)	0.001	0.0001	0.103
19	Fruit crops	1 (8)	0.001	0.0001	0.131
20	Oil crops	1 (7)	0.030	0.0050	0.920
21	Rapeseed	1 (7)	0.035	0.0056	0.910
22	Oil palm	1 (7)	0.015	0.0031	0.940
23	Soybean	2 (7)	0.062	0.0050	0.850
24	Groundnut	1 (7)	0.057	0.0042	0.942
25	Sunflower	1 (7)	0.034	0.0045	0.920
26	Sesameseed	1 (7)	0.033	0.0056	0.940
27	Coconut	1 (8)	0.002	0.0004	0.896
28	Cocoa	1 (8)	0.014	0.0026	1
29	Coffee	1 (8)	0.024	0.0022	1
30	Tea	1 (8)	0.078	0.0150	0.525
31	Tobacco	1 (8)	0.003	0.0006	0.21
32	Cotton	1 (8)	0.029	0.0053	0.92
33	Fiber crops	1 (8)	0.081	0.0095	1
34	Rubber	1 (8)	0.000	0.0000	1

[a] 1 = upland cops; 2 = legumes; 3 = rice; crop number 35 is grass, which is crop group 4 but not considered here. The numbers in brackets refer to the grouping used in Table SI4.

Table SI3. List of fodder crops[a] included in the data.

1	Maize for forage and silage	Cattle
2	Sorghum for forage and silage (	Cattle
3	Rye grass for forage and silage (	Cattle
4	Grasses not elsewhere specified for forage and silage	Cattle
5	Clover for forage and silage	Cattle
6	Alfalfa for forage and silage	Cattle
7	Green oilseeds for silage	Cattle
8	Leguminous for silage	All
9	Cabbage for fodder	All
10	Turnips for fodder	All
11	Beets for fodder	All
12	Carrots for fodder	All
13	Swedes for fodder	All
14	Forage products	All
15	Vegetables roots fodder	All

[a] The class of pumpkins for fodder is excluded because the volume of production seems unrealistic (L. Lassaletta, personal communication, March 2015).

Table SI4. N and P as a fraction of dry matter of crop residues for 8 crop groups.

#[a]	N fraction	P fraction
1.Temperate cereals	0.0067	0.0008
2. Rice	0.0067	0.0009
3. Maize	0.0110	0.0020
4. Tropical cereals	0.0080	0.0009
5. Pulses	0.0118	0.0011
6. Roots and tuber crops	0.0264	0.0029
7. Oil crops	0.0110	0.0027
8. Other crops	0.0117	0.0016

[a] for grouping see Table SI2. Based on data available from INRA/CIRAD/ Association Française de Zootechnie/ FAO [4].

Table SI5. Crop residues used as animal feed in different world regions, and amount of N and P withdrawal from the soil-plant system around 2010.

Region	Crop residues used as animal feed		
	Tg dry matter[a]	Tg P	Tg N
Europe and Russia	27	0.03	0.2
Oceania	1	0.00	0.0
North America	25	0.03	0.2
South America	30	0.03	0.3
Eastern Asia	48	0.05	0.4
South East Asia	37	0.04	0.3
South Asia	259	0.28	2.3
North Africa	17	0.02	0.1
sub-Saharan Africa	128	0.14	1.2
Total	572	0.629	5.1

[a] Source: Herrero et al. [5].

Table SI6. Excretion rates for the different animal categories.

Animal category	Excretion in kg head^{-1} year^{-1} N	P
1. Beef cattle	60[a]/50[b]/40[c]	10.5[a]/8.7[b]/7.0[c]
2. Dairy cattle	130[a]/100[b]/60[c]	22.7[a]/17.5[b]/10.5[c]
3. Buffaloes	45	7.9
4. Pigs	11	1.8
5. Poultry	0.5	0.1
6. Sheep and goats	10	1.5
7. Horses	50[d]/45[c]	7.3[d]/6.5[c]
8. Asses	30	4.4
9. Mules	30	4.4
10. Camels	55	8.0

[a] Canada, USA, Japan;
[b] = OECD Europe;
[d] = Canada, USA, OECD Europe, Japan;
[c] = all other countries.

Table SI7. Comparison of global N budget terms from different sources for the year 1995.

Year 1995	This paper	Lassaletta et al. [6]	Bodirsky et al. [7]	Smil [8]	Sheldrick et al. [9]
			Tg N yr^{-1}		
Total fertilization	140	126	137	138	123
Uptake	63	56	63	60	63
Surplus	77	70	74	78	60
Manure	29	21	24	18	25
Synthetic fertilizer	74	72	78	78	78
Biological fixation	22	24	20	22	n.d.
Deposition	9	15	15	20	20

Table SI8. Comparison of global N budget terms from different sources for the year 2009.

	This paper	Lassaletta et al. [6]
	Tg N yr^{-1}	
Total fertilization	167	163
Uptake	82	75
Surplus	85	88
Manure	34	26
Synthetic fertilizers	89	97
Biological fixation	27	30
Deposition	17	10

Table SI9. Comparison of global P budget terms from different sources for the year 2000 and 2013 for croplands

Budget term	Year 2000		Year 2013	
	This study	MacDonald et al. [10]	This study	Chen et al. [11]
	Tg P yr^{-1}			
Fertilizer P applications	14	14	17	20
Manure P applications	6	10	8	7
Crop P removal	11	12	15	12
Agronomic balance	9	11	9	14

SI Figures

Figures SI5a-e have been prepared with the graphical programme XY [12]

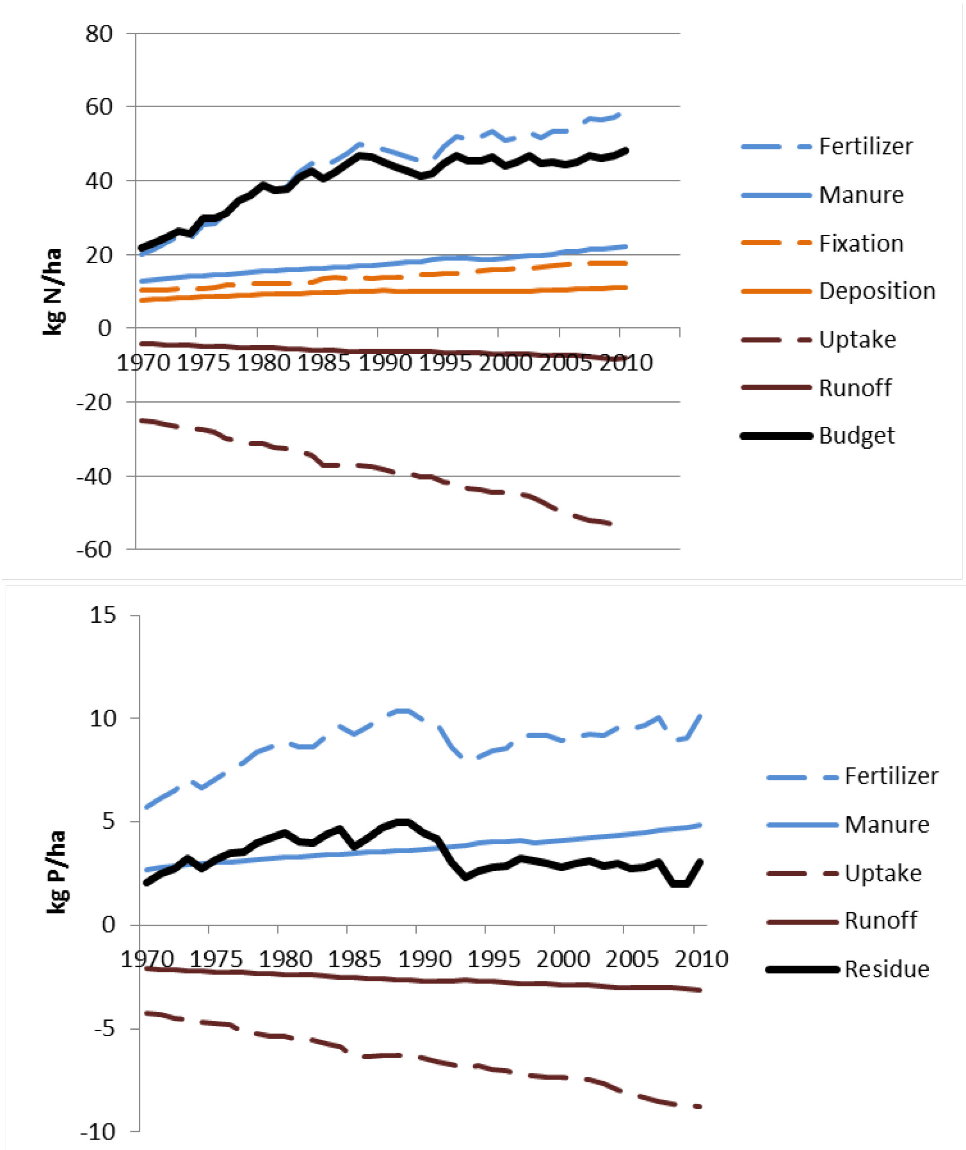

Figure SI1. Global balance terms and budget/residue for N (top) and P (bottom). Data for regional N and P soil budget terms are in the SI_datafiles.

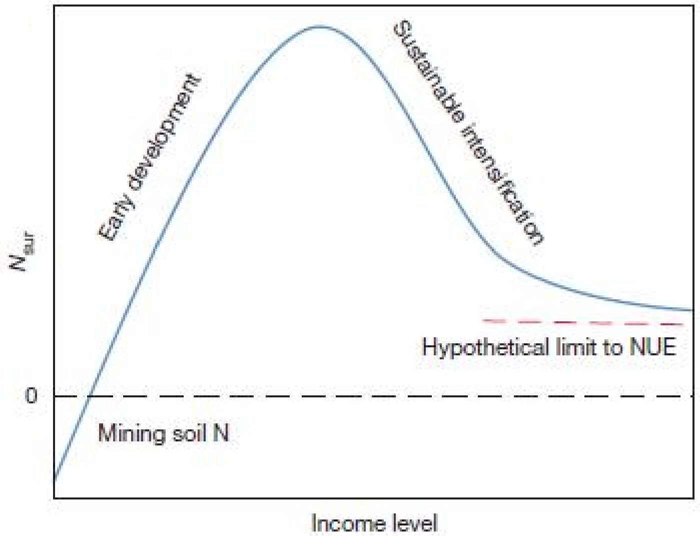

Figure SI2. Environmental Kutznets curve showing idealized relationship between income level and N surplus (N_{sur}). Source: Zhang et al. [2].

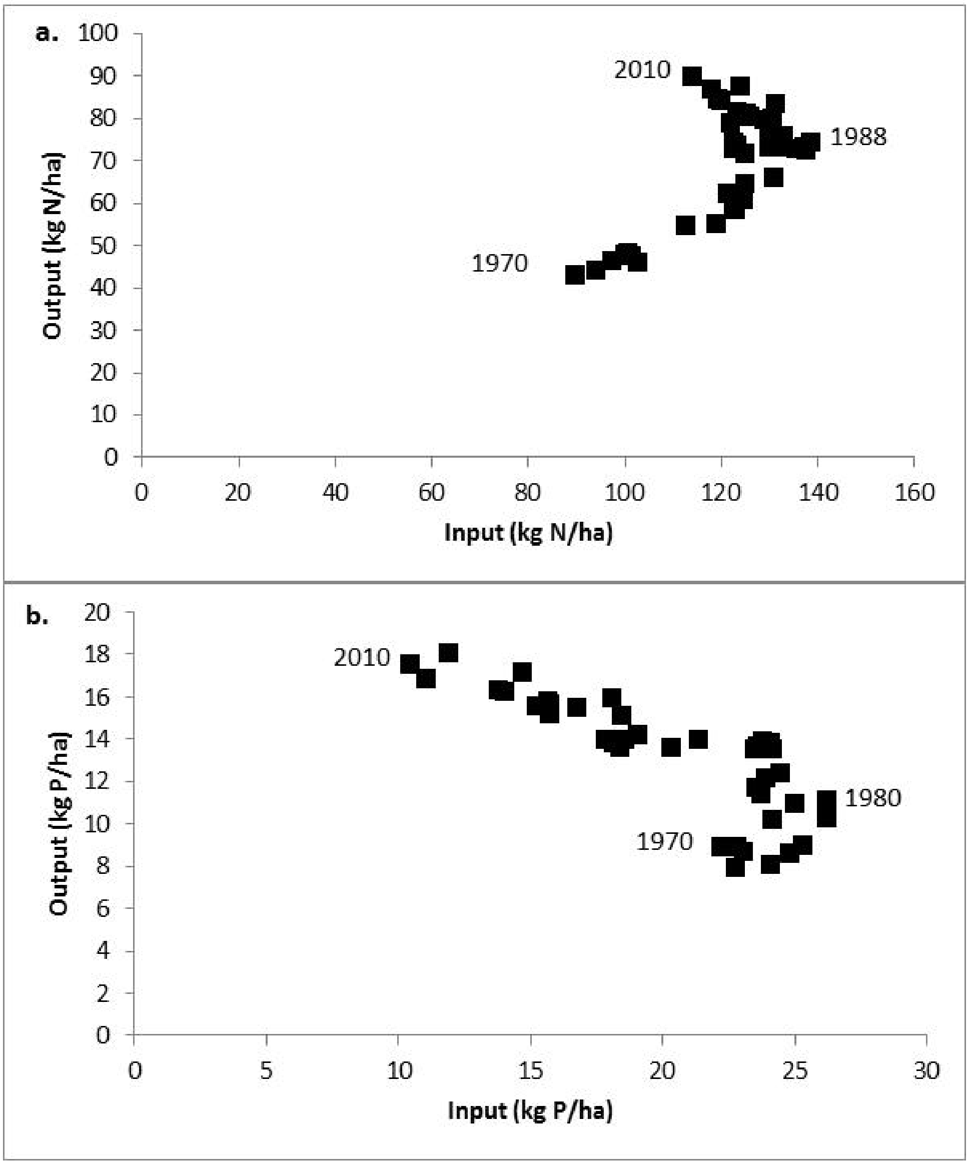

Figure SI3. Relationship between a) N inputs and N uptake and b) P inputs and P uptake (bottom panel) by crops in Western Europe. The hysteresis of N inputs can be explained by increased N use efficiencies, while that of P inputs is because of the accumulation and use of residual P.

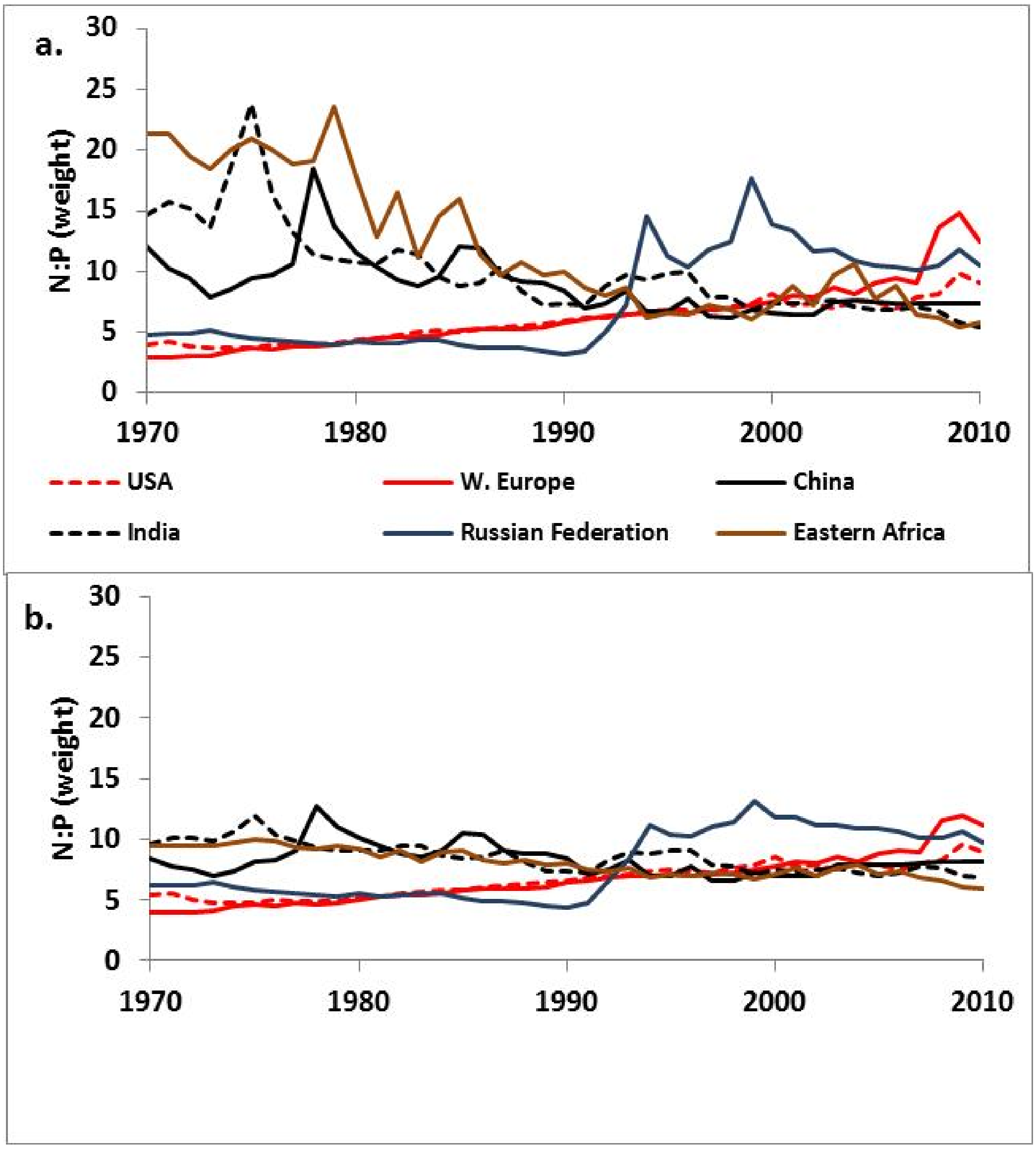

Figure SI4. N:P ratio (weight basis) of a) new N inputs (N fertilizer, N fixation) and new P inputs (P fertilizer); and of b) total N and P inputs.

a. Manure

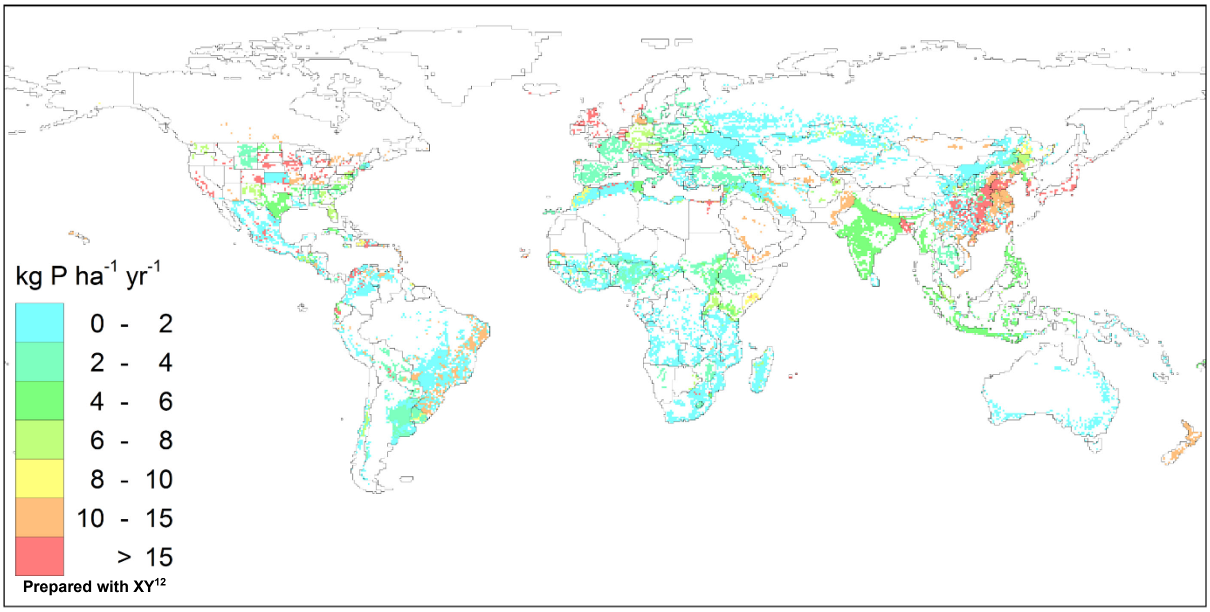

Figure SI5a. Global 0.5 by 0.5 degree distribution of manure P inputs for 2010.

b. Fertilizer

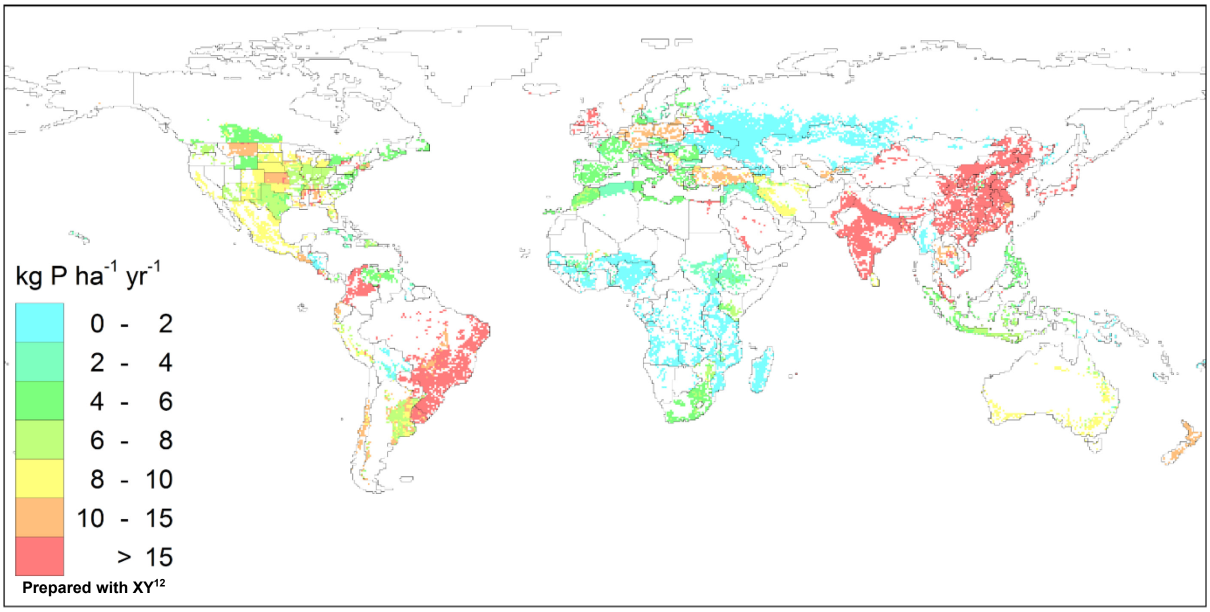

Figure SI5b. Global 0.5 by 0.5 degree distribution of synthetic fertilizer P inputs for 2010.

c. Surface runoff

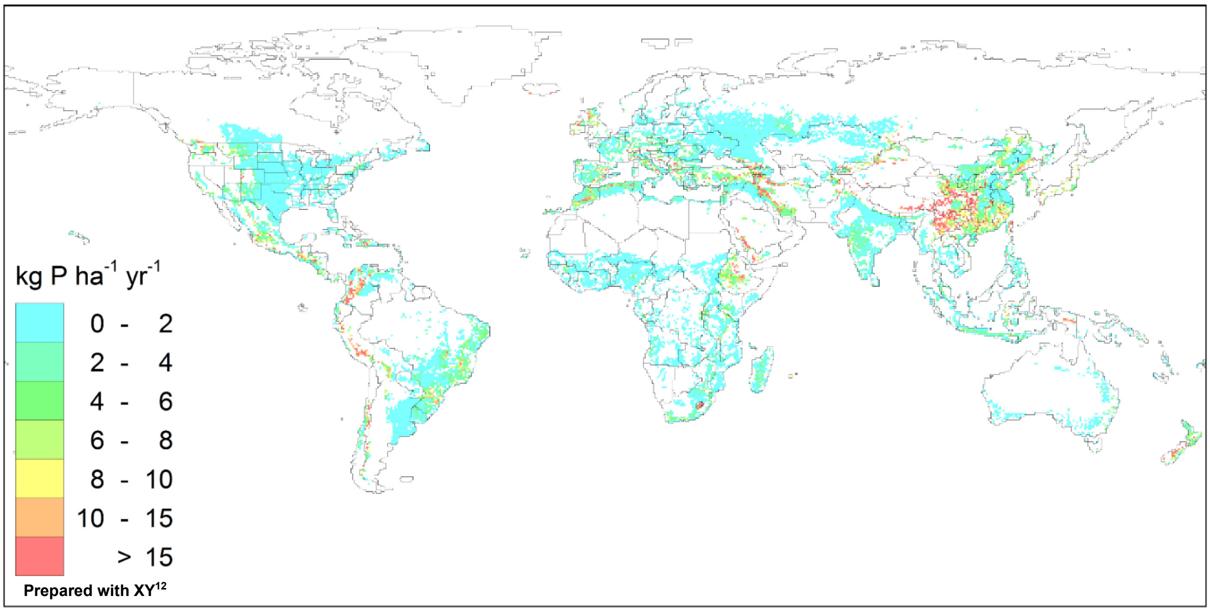

Figure SI5c. Global 0.5 by 0.5 degree distribution of surface runoff P loss for 2010.

d. Weathering

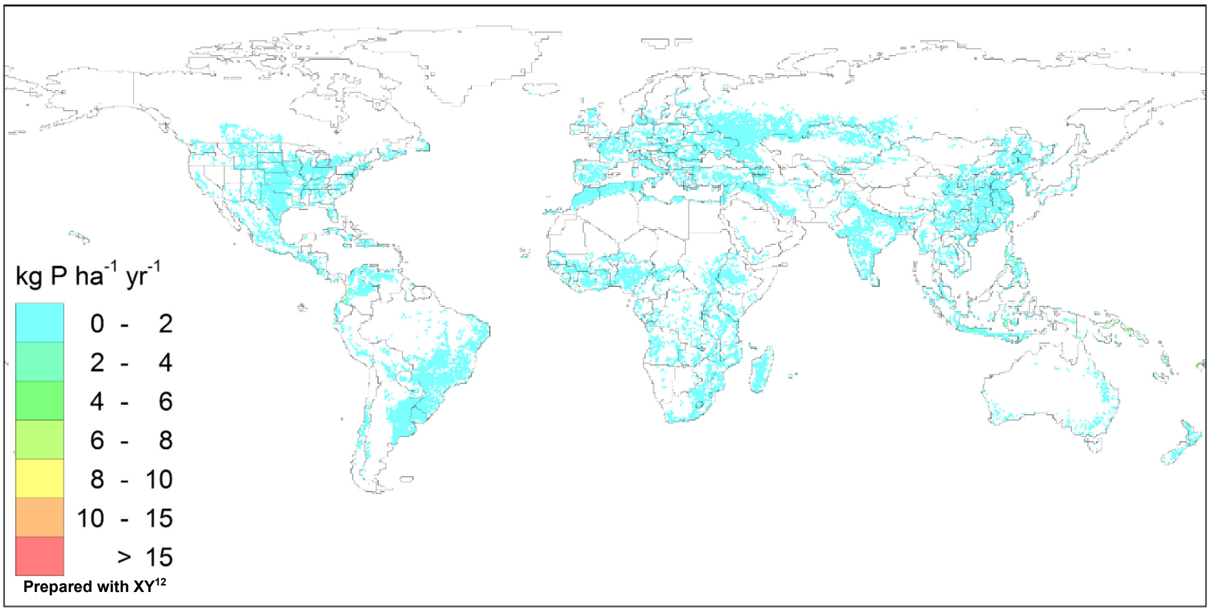

Figure SI5d. Global 0.5 by 0.5 degree distribution of weathering P losses from soil landscapes for 2010.

Figures SI5a-e have been prepared with the graphical programme XY [12]

e. Uptake

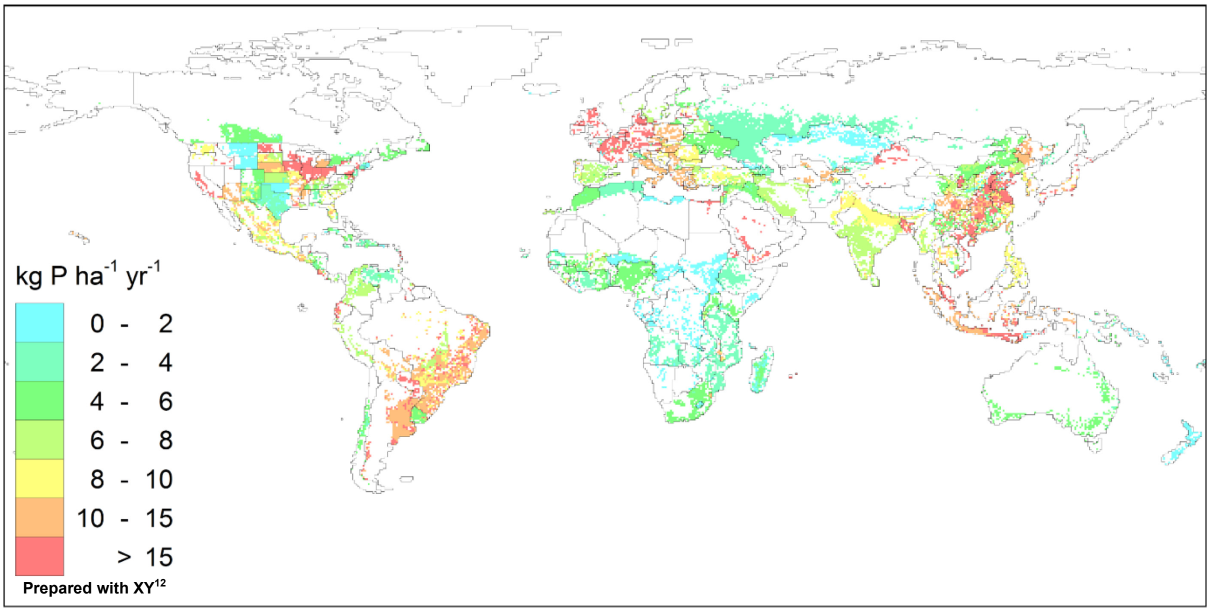

Figure SI5e. Global 0.5 by 0.5 degree distribution of crop P uptake for 2010.

Literature

1 Hunter, J. D. Matplotlib: A 2D graphics environment. *Computing in Science and Engineering* **9**, 90-95 (2007).

2 Zhang, X. *et al.* Managing nitrogen for sustainable development. *Nature* **528**, 51-59, DOI:10.1038/nature15743 (2015).

3 Stehfest, E., Van Vuuren, D. P., Kram, T. & Bouwman, A. F. *Integrated Assessment of Global Environmental Change with IMAGE 3.0. Model description and policy applications*. 366 (PBL Netherlands Environmental Assessment Agency (http://themasites.pbl.nl/models/image/index.php/Main_Page), 2014).

4 INRA, CIRAD, Association_Française_de_Zootechnie & FAO. *Feedipedia: An on-line encyclopedia of animal feeds. http://www.feedipedia.org/, accessed 6 April 2016.*, 2016).

5 Herrero, M. *et al.* Biomass use, production, feed efficiencies, and greenhouse gas emissions from global livestock systems. *Proceedings of the National Academy of Sciences of the United States of America* **110**, 20888-20893, DOI:10.1073/pnas.1308149110 (2013).

6 Lassaletta, L. *et al.* Nitrogen use in the global food system: past trends and future trajectories of agronomic performance, pollution, trade, and dietary demand. *Environmental Research Letters (Accepted)* (2016).

7 Bodirsky, B. L. *et al.* N2O emissions from the global agricultural nitrogen cycle-current state and future scenarios. *Biogeosciences* **9**, 4169-4197, DOI:10.5194/bg-9-4169-2012 (2012).

8 Smil, V. Nitrogen in crop production: An account of global flows. *Global Biogeochemical Cycles* **13**, 647-662 (1999).

9 Sheldrick, W., Syers, J. K. & Lingard, J. Contribution of livestock excreta to nutrient balances. *Nutrient Cycling in Agroecosystems* **66**, 119-131 (2003).

10 MacDonald, G. K., Bennett, E. M., Potter, P. A. & Ramankutty, N. Agronomic phosphorus imbalances across the world's croplands. *Proceedings of the National Academy of Sciences of the United States of America* **108**, 3086-3091, DOI:10.1073/pnas.1010808108 (2011).

11 Chen, M. & Graedel, T. E. A half-century of global phosphorus flows, stocks, production, consumption, recycling, and environmental impacts. *Global Environmental Change* **36**, 139-152, DOI:10.1016/j.gloenvcha.2015.12.005 (2016).

12 Van Heerden, C. & Tiktak, A. Het grafisch programma XY. Een programma voor visualisatie van de resutaten van rekenprogramma's. SOTRAS deelrapport nr. 2. Report No. 715501002, 82 (Rijksinstituut voor Volksgezondheid en Mileiuhygiëne, Bilthoven, The Netherlands, 1994).
