# Supplementary figures and images for: Lessons from temporal and spatial patterns in global use of N and P fertilizer on cropland
Source: Sci Rep. 2017 Jan 13;7:40366. doi: 10.1038/srep40366 (PMC5234009; doi:10.1038/srep40366)

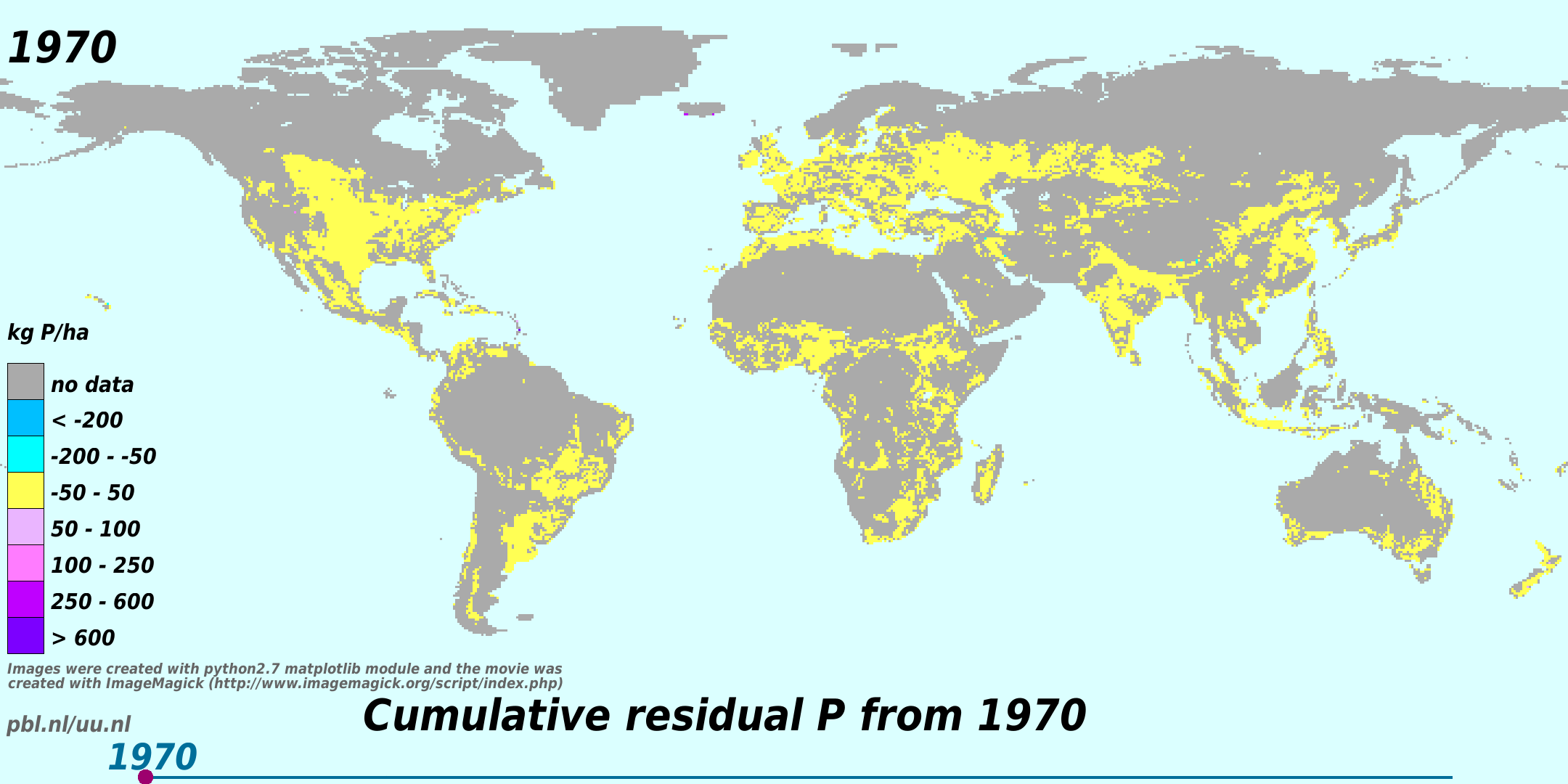

Supplement: Supplementary Movie [file srep40366-s2.gif]
